# Silica Xerogel Doped with Iron(III) as Sensor Material for Salicylhydroxamic Acid Determination in Urine

**DOI:** 10.3390/gels7030143

**Published:** 2021-09-16

**Authors:** Maria A. Morosanova, Vitaliy A. Kovalev, Elena I. Morosanova

**Affiliations:** Analytical Chemistry Division, Chemistry Department, Lomonosov Moscow State University, 119234 Moscow, Russia; m.a.morosanova@gmail.com (M.A.M.); conc.lab.student@gmail.com (V.A.K.)

**Keywords:** sensor materials, silica xerogels, salicylhydroxamic acid determination, biological samples, urine

## Abstract

Salicylhydroxamic acid (SHA) is used as antimicrobic medicine and its concentration has to be monitored in urine. For the first time, silica xerogels doped with iron(III) have been proposed as sensor materials for SHA determination in biological samples. Three xerogels with iron(III) content in the range of 0.04–1.74% wt have been synthesized. BET surface area of these xerogels has varied in the range of 696–529 m^2^/g and total pore volume has varied in the range of 0.92–0.23 cm^3^/g. Complex formation between immobilized iron(III) and salicylhydroxamic acid has been investigated with solid phase spectrophotometry and IR spectroscopy. Orange-brown iron(III)-SHA complex with 1:1 stoichiometry is formed at pH 1–4 with half-reaction time of 17 min. Silica xerogel doped with 0.33% wt iron(III)) has been used as sensor material for SHA solid phase spectrophotometric determination (LOD 1.4 mg/L (*n* = 3), analytical range 4–230 mg/L). Proposed sensor material has been applied for SHA determination in biological samples of synthetic and human urine. The proposed procedure is characterized by a good level of accuracy (recovery values 97–120%) and precision (RSD values 4–9%) and can be recommended for pharmacokinetic–pharmacodynamic studies of hydroxamic acid-based medications.

## 1. Introduction

Hydroxamic acids (HA) are well-known for their outstanding complexation properties: complexes with 19 metals are known. The most prominent complexes are formed with iron(III) and they are also often found in nature to increase the bioavailability of insoluble iron compounds [1].

The biological and medical importance of HA is well recognized in processes such as microbial iron transport, the hem-dependent prostaglandin-H synthase, inhibition of the nickel-dependent urease enzymes, and the zinc-dependent matrix metalloproteinases [2]. HA are outstanding zinc chelating compounds that can be used to design potent and selective metalloenzyme inhibitors in various therapeutic areas [3]. Several HA have been reported to possess antibacterial or antiviral properties. The application of HA in the non-infectious diseases include inhibition of glutamate carboxypeptidase II in neuropathic pain, inhibition of insulin-degrading enzyme in type 2 diabetes, and inhibition of matrix metalloprotease in fibrinolysis control [3].

Three HA—salicylhydroxamic acid (SHA), suberanilohydroxamic acid (SAHA), and deferoxamine (DFA)—are already commonly used in medicine [1,4,5]. However, these medications have been linked with various acute and chronic side effects [6]. The determination of HA is a task of great importance. Efficient and selective analytical methods for HA determination in plasma and urine samples are required for both dose regulation and clinical studies (pharmacokinetic–pharmacodynamic parameters, efficiency of the drugs) [6,7]. Up to date, several analytical methods have been used for HA determination: chromatographic [5,6,7,8,9], electrochemical [10,11], and spectrophotometric [12,13]. Some of the procedures are based on complexes formation between HA and metals [9,13]. No on-site procedures, including the use of sensor materials, have been described to date for HA determination.

On-site determination of potentially health-endangering substances, such as HA-based medications, is required. The popular approach to simple and reliable on-site analysis is using chemically active sensor materials. Silica xerogels with molecular imprinting or with immobilized reagents are widely used for biological analytes determination [14,15,16,17,18,19]. Their main advantages are chemical inertness, mechanical rigidity, and remarkable stability over time. Tunable pore size distribution and physicochemical properties make them an ideal basis for molecularly imprinted materials [14,15] and selectively permeable membranes [16,17]. Silica and silica–titania sol–gel xerogels present a stable matrix for the immobilization of various recognition elements (DNA, antibodies, enzymes [18,19], analytical reagents [20,21]) for biological analytes determination. Silica xerogels have not been used for HA determination in biological samples yet.

The aim of the present work was to design new sensor material for simple SHA determination in biological samples using silica xerogels doped with iron(III).

## 2. Results and Discussion

### 2.1. Synthesis of Silica Xerogels Doped with Iron(III)

Salicylhydroxamic acid (SHA) is used as a therapeutic agent for urolithiasis and as a trypanocidal and antitubercular agent [10], and its concentration has to be monitored in urine. The goal of this work was to create sensor materials for simple SHA determination in urine. The material should change its color when contacting with SHA. Since SHA is known for forming colored complexes with iron(III), we decided to prepare silica xerogel doped with iron(III) for that purpose. Recently, we proved that iron(III) included in xerogels retains the ability to form complexes [22].

In the present work we studied the interaction silica xerogels doped with iron(III) as the recognition element for SHA and chose the conditions for the complex forming reaction between immobilized iron(III) and SHA.

**Figure 1:**
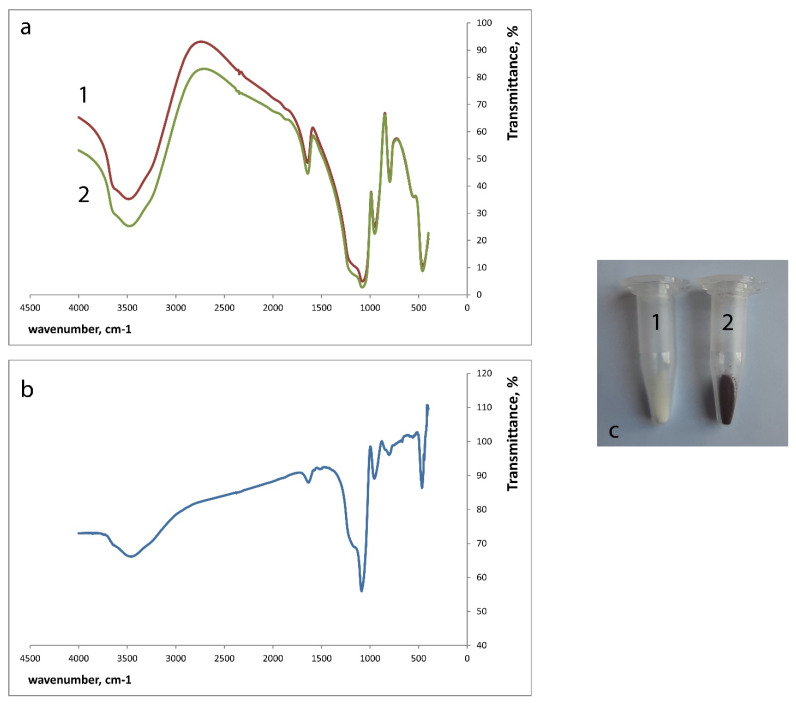
SiFe10 xerogel interaction with 5.0 × 10^−3^ M salicylhydroxamic acid (SHA) (pH 3.3, time of contact 30 min), 1—SiFe10 xerogel, 2—SiFe10 xerogel after the interaction with SHA. (**a**)—IR spectra of silica xerogels against KBr, (**b**)—iFe10 xerogel after the interaction with 5.0 × 10^−3^ M SHA IR spectrum against SiFe10 xerogel, (**c**)—coloration of SiFe10 xerogel.

Silica xerogels doped with iron(III) were synthesized by adding iron(III) chloride water solutions to the hydrolyzing mixture using our recently proposed procedure [22], with modifications in iron content. The content of the recognition element in the sensor material influences the sensitivity of the determination. In our previous work we have studied silica–titania xerogels with 1.0 × 10^−3^ M and 1.0 × 10^−1^ M iron(III) concentration in the sol. While the first xerogel was successfully applied as functional sensor material, the increase of iron(III) content led to the loss of its complex forming ability [22]. The influence of iron(III) content in silica xerogels on sensor material properties has not been studied before and became a subject of this study. Three different xerogels were synthesized: SiFe1, SiFe10, and SiFe50-silica xerogels doped with 1.0 × 10^−3^ M, 1.0 × 10^−2^ M, and 5.0 × 10^−2^ M iron(III) in sol (Table 1). SiFe1 was white, SiFe10 was very light yellow, and SiFe50 was brown. The brown color seems to be caused by the formation of iron oxide with the sufficient iron(III) content and the rapid evaporation of water during the drying of xerogel. The amount of iron(III) in the xerogels was calculated as the difference between the amount added to the sol and the amount found in the washing water fractions (Table 1). The increase of iron(III) concentration in sol led to the proportional increase of iron(III) content in the xerogel (Pearson’s correlation coefficient equals 0.9999), which allows the synthesis of the xerogel with predetermined iron(III) content. IR spectrum of SiFe10 (Figure 1a) shows a lot of hydroxyl groups in the xerogel. There is a wide absorbance band at 3500 cm^−1^ that corresponds to a bridging hydroxyl group. The molecular water bending vibration band (1640 cm^−1^) is also observed. 1090–1100 cm^−1^ band belongs to the Si-O stretching vibration and 800 cm^−1^ band belongs to the deformation vibrations of Si-O-Si band. These data allow suggesting that immobilized iron atoms are coordinated by the silanol groups of the matrix and by the adsorbed water in the xerogel.

The textural characteristics of the xerogels are very important for the analytical applications, so in the present work the influence of iron(III) content on the textural characteristics of silica xerogels doped with iron(III) was investigated (Table 1).

The increase of iron(III) content in the xerogels led to a decrease in BET surface area and average pore diameter. The micropore area and micropore volume, on the contrary, increased when the iron(III) content increased tenfold (SiFe10). However, further increase of iron(III) content (SiFe50) showed the decrease of the micropore area and micropore volume, which may also signify the formation of iron oxide in the pores of the xerogel similarly to [22,23].

### 2.2. Interaction of Iron(III) Immobilized in Silica Xerogels with Salicylhydroxamic Acid

In solution, iron(III) and SHA form colored complexes of various composition. The dissociation constants of salicylhydroxamic acid equal 6.73 and 9.15 [24], which allows it to be described as H_2_L in the complex forming reaction in acidic media. In highly acidic media soluble 1:1 complex is formed [24], and there are also data on insoluble 1:2 complexes [24] and 1:3 complexes [2]. Only carbohydroxamic functional group takes part in the coordination of iron(III) in the complex with SHA in solution, one proton being split off and the five membered chelate ring formed [24]. However, in the interaction of iron(II) with SHA on cassiterite surface the coordination by N atoms is also observed [25]. The interaction of SHA with metals demonstrates results in the N–H (3100 cm^−1^) and O–H (2900 cm^−1^) stretching adsorption bands disappearance and the formation of metal–O (557–668 cm^−1^) and metal–N (412–485 cm^−1^) bands [25].

In the present work, we studied the interaction of silica xerogels doped with iron(III) with salicylhydroxamic acid (SHA) as a model hydroxamic acid. The contact of the silica xerogels doped with iron(III) with SHA resulted in the SiFe1 and SiFe10 xerogels changing color to orange–brown (Figure 1c). SiFe50 xerogel did not change color after contact with SHA. The increase of iron(III) amount led to the loss of its ability to form complexes with SHA, probably due to polymerization of iron atoms as it was observed before [22].

Complex formation between immobilized iron(III) and SHA has been investigated with IR spectroscopy and solid phase spectrophotometry.

IR spectra of the xerogels were recorded using the incorporation of the ground xerogels in KBr discs. The spectra of blank SiFe10 xerogel and SiFe10 xerogel after the interaction with 5.0 × 10^−3^ M SHA against KBr (Figure 1a) demonstrate wide rounded peaks, which mean that xerogels are characterized by amorphous state and contain much water. The IR spectra for SiFe10 xerogel before and after the interaction with SHA looked similar.

The differences between SiFe10 xerogel and SiFe10 xerogel after the interaction with 5.0 × 10^−3^ M SHA became more easily observed when we recorded them one against another (Figure 1b): the absorption bands can be observed at 1640, 1090, 960, 850, and 470 cm^−1^. Fe-N absorption band (470 cm^−1^) formation can be observed, but not the Fe-O band, which can be explained by the background signal of many Fe-O bonds between the silica matrix, absorbed water, and iron(III).

**Figure 2:**
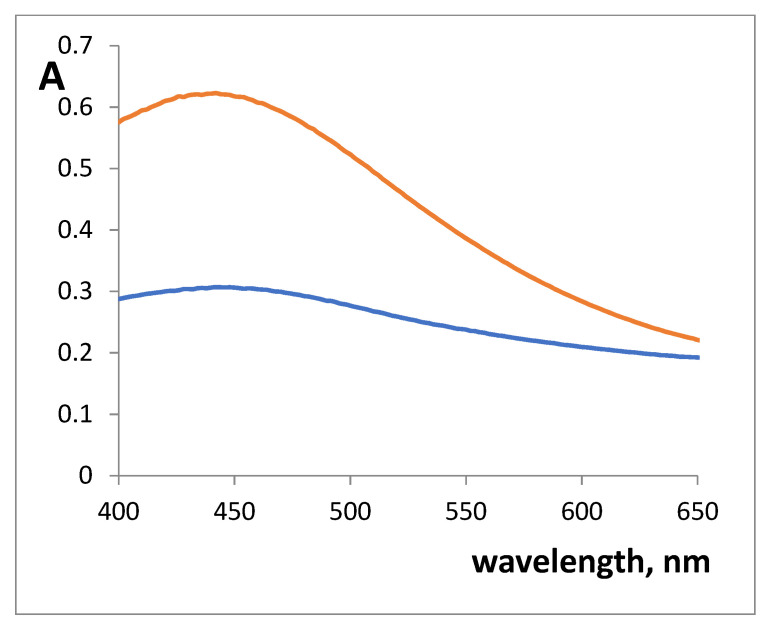
Absorption spectra of silica xerogels doped with iron(III) absorbance after the interaction with 3 × 10^−3^ M salicylhydroxamic acid (SHA). m/V = 0.10 g/5.0 mL, pH 3.3, time of contact is 30 min. blue—SiFe1, red—SiFe10.

The UV-vis absorption spectra of colored SiFe1 and SiFe10 xerogels, obtained after contact with 3.0 × 10^−3^ M SHA, are given in Figure 2. Xerogels absorption maximum was observed at 450 nm. Iron(III) complex with SHA in solution is characterized by 530–535 nm absorption maximum [24]. Such a difference in the absorption maxima has been observed for colored complexes in the xerogels compared to the complexes in the solution.

**Figure 3:**
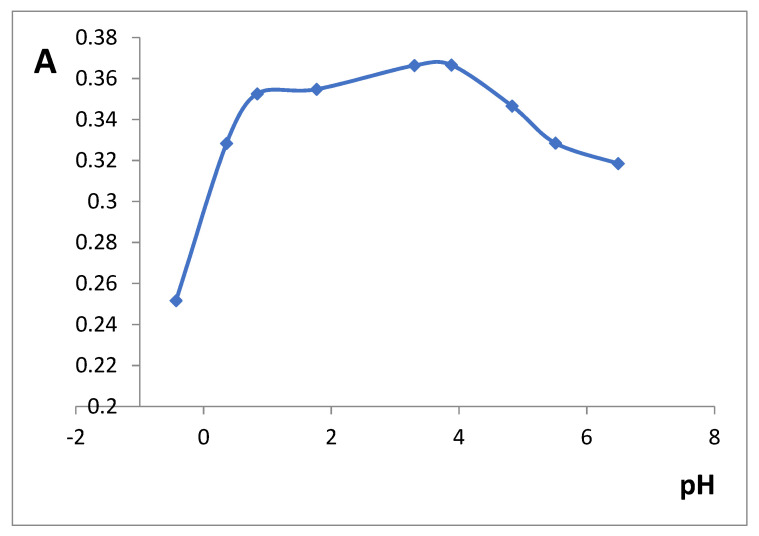
The effect of pH on silica xerogel doped with iron(III) (SiFe1) absorbance after the interaction with 3 × 10^−3^ M SHA. λ 450 nm, m/V = 0.10 g/5.0 mL, time of contact is 30 min.

The influence of pH on the iron(III)-SHA complex formation was studied in the range of 0–7 pH (Figure 3). Maximal xerogel absorbance values were observed in a rather wide range (pH 1–4) and we chose pH 3.3 for the further experiments. In solution, iron(III)–SHA complex is only soluble at pH 0–2 [24].

The interaction of SHA (ligand, H_2_L) with iron(III), immobilized in silica xerogel and coordinated by xerogel silanol groups, can be described by the following equation:

(1) $$\bar{\mathrm{Fe}(\mathrm{OH})m}+\;\mathrm{nH}2L\;={\bar{\mathrm{Fe}(\mathrm{OH})m-n(\mathrm{HL})}}_{n}+\mathrm{nH}2O$$

The complex stoichiometry (n) and the equilibrium constant (K_eq_) were determined with the help of the equilibrium shift method [26]. The equilibrium constant of the reaction (1) is defined in the Equation (2). The logarithmic form of this Equation (3) turns this equation into a linear one.

(2) $$\mathrm{Keq}=[{\bar{\mathrm{Fe}(\mathrm{OH})m-n(\mathrm{HL})}}_{n}]/([\bar{\mathrm{Fe}(\mathrm{OH})m}]\cdot {\left[H2L\right]}_{}^{n})$$

(3) $$\mathrm{lg}([{\bar{\mathrm{Fe}(\mathrm{OH})m-n(\mathrm{HL})}}_{n}]/[\bar{\mathrm{Fe}(\mathrm{OH})m}])=lgKeq+n\cdot \mathrm{lg}[H2L]$$

**Figure 4:**
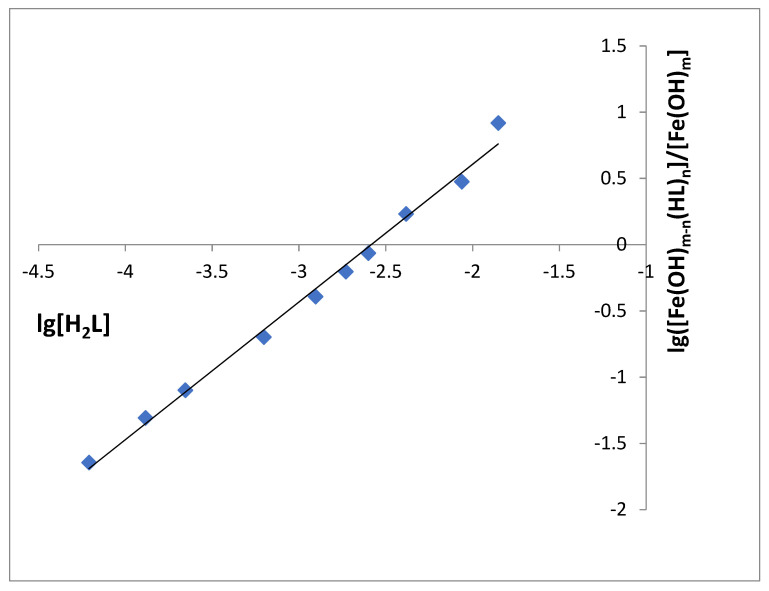
The dependence of lg([Fe(OH)_mm−nn_(HL)_n_]/[Fe(OH)_m_]) on lg[H_2_L]. λ 450 nm, pH 3.3, m (SiFe1)/V = 0.10 g/5.0 mL, time of contact is 60 min.

[Fe(OH)_m−n_(HL)_n_]/[Fe(OH)_m_] is calculated as A_i_/(A_ex_ − A_i_), where A_i_ is the xerogel absorbance after the contact with SHA and A_ex_ is the xerogel’s absorbance after the contact with the excess of SHA. [H_2_L] is the residual concentration of SHA in solution after this reaction. lg([Fe(OH)_m−n_(HL)_n_]/[Fe(OH)_m_]) is a linear function of lg[H_2_L] (3), so the parameters of this function (n and K_eq_) can be calculated using simple linear regression analysis of the experimental data (Figure 4, Table S1). The linear approximation (lg([Fe(OH)_m−n_(HL)_n_]/[Fe(OH)_m_]) = 1.04·lg[H_2_L] + 2.69, R^2^ = 0.9924) gives the complex stoichiometry of 1:1 and the equilibrium constant of 485 M^−1^. In solution iron(III)–SHA complex can have 1:1, 1:2, and 1:3 stoichiometry, and this difference can be explained by steric hindrance of the second and third ligand introduction. Such effects had been observed for many metal–ligand complexes in the xerogel phase before [27].

**Figure 5:**
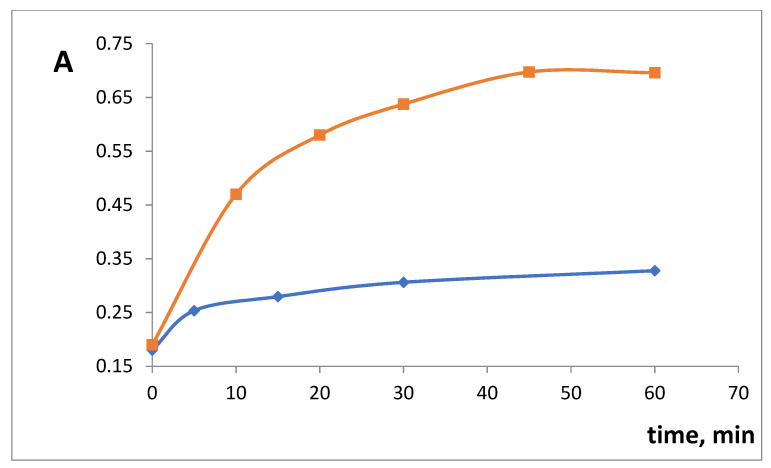
The kinetics of the interaction of silica xerogels doped with iron(III) absorbance after the interaction with 3 × 10^−4^ M salicylhydroxamic acid (SHA). λ 450 nm, m/V = 0.10 g/5.0 mL, pH 3.3. blue—SiFe1, red—SiFe10.

The influence of time of xerogel contact with SHA on the complex formation was studied. For xerogels SiFe1 and SiFe10, the equilibrium was reached at 60 min after the reaction start (Figure 5). An earlier developed approach [28] was used to calculate half-reaction periods (T_1/2_): 17 min for SiFe1 and 16 min for SiFe10. The increase of iron(III) content did not influence the reaction speed, most probably due to the above-mentioned steric factor being more important than the iron(III) content.

SiFe1 and SiFe10 xerogels were shown to be able to form colored complex with SHA and they were chosen for the further evaluation as sensor materials for SHA determination.

### 2.3. Analytical Application

Chosen conditions (450 nm, pH 3.3) were used to develop the analytical procedures of solid phase spectrophotometric determination of SHA in urine samples. Calibration curves were constructed using xerogel absorbance at 450 nm as analytical signal. The interaction of xerogels SiFe1 and SiFe10 with SHA was studied in the 1.0 × 10^−6^ M–1.0 × 10^−2^ M range and the calibration curve slopes were determined (Table 2). The limit of detection (LOD) was calculated as 3 × standard deviation of the blank divided by the slope value. Limit of quantification (LOQ) was calculated as 3·LOD. Since the time of analysis is an important parameter, we compared the calibration curves at 20 min and at 60 min for SiFe1 xerogel. This increase of the reaction time led to the increase of the determination sensitivity, however, the effect was not very pronounced. We chose 60 min of the reaction time for our experiments, but if needed the analysis can be performed in 20 min with a small loss in sensitivity. The increase of iron(III) content was accompanied with almost tenfold increase of the sensitivity (slope value), so SiFe10 was chosen for further experiments.

We have studied the interferences of some substances present in the biological samples: the samples containing 1.0 × 10^−4^ M of SHA and varying amounts of the interfering substance were prepared. SHA concentration in these samples was calculated using the calibration curve. The interference threshold for each interfering substance was defined as the concentrations giving 10% or less error in the determination of SHA: up to 5.0 × 10^−3^ M salicylate, 5.0 × 10^−4^ M ascorbic acid, and 700 mg/L albumin did not interfere the determination of 1.0 × 10^−4^ M of SHA (Table S2).

The proposed procedure was applied to SHA determination in the spiked samples of synthetic and human urine (Table 3). Recoveries and relative standard deviation values demonstrate a good level of accuracy and precision of the proposed analytical procedure, as well as the applicability to the medical analyses.

We compared the LOD values of the proposed procedure with other solid phase spectroscopic procedures for HA determination (Table 4). The advantages of the proposed procedure are the simplicity and have the possibility of field applications. Silica xerogels doped with iron(III) are very stable sensor materials (at least 6 months stable [22]). Sensor materials based on xerogels can be easily applied to the complex biological samples, because high interference thresholds are provided by the extraction of the analyte to the solid phase and the following measurement of the solid phase absorbance. Based on high stability and simplicity of application, silica xerogels doped with iron(III) have a great potential for the determination of hydroxamic acids in such areas as the pharmacokinetic studies, pharmaceutical quality control, or medical analysis.

## 3. Conclusions

In order to design the sensor material for salicylhydroxamic acid (SHA) determination, we have prepared silica xerogels doped with various content of iron(III) (0.04–1.74% wt). The increase of iron(III) content has led to a decrease in BET surface area and average pore diameter. The ability of immobilized iron(III) to form colored 1:1 complexes with SHA has been demonstrated for the first time. The increase of iron(III) content led to the increase of xerogel absorbance after the interaction with SHA.

Silica xerogel doped with iron(III) (0.33% wt) has been chosen as the new sensor material for the determination of SHA in urine samples. Solid phase spectrophotometric procedure has been proposed for SHA used as a model hydroxamic acid (LOD 1.4 mg/L, analytical range 4.2–230 mg/L). We have studied the interferences of some substances present in the biological samples: 5.0 × 10^−3^ M salicylate, 5.0 × 10^−4^ M ascorbic acid, and 700 mg/L albumin did not interfere the determination. Proposed solid phase spectrophotometric procedure has been applied for SHA determination in urine samples. The proposed procedure is characterized by a good level of accuracy (recovery values 97–120%) and precision (RSD values 4–9%), and can be recommended for the determination of hydroxamic acids in such areas as the pharmacokinetic studies, pharmaceutical quality control, or medical analysis.

## 4. Materials and Methods

### 4.1. Reagents and Apparatus

Salicylhydroxamic acid (SHA), ascorbic acid, sodium salicylate, iron(III) chloride hexahydrate, tetraethyl orthosilicate, trisodium citrate, ammonium chloride, urea, and creatinine were purchased from Acros Organics (Carlsbad, CA, USA). Sodium chloride, sodium sulfate, and potassium chloride were purchased from Merck (Kenilworth, NJ, USA). Calcium chloride, magnesium chloride, sodium oxalate, and monopotassium phosphate were purchased from Chimmed (Moscow, Russia). Hydrochloric acid was purchased from Sigma Tec (Moscow, Russia). Ethanol (96%) was purchased from Baum-Lux (Moscow, Russia). Albumin and glucose were purchased from PanEco (Moscow, Russia). All the reagents were of analytical grade.

SHA stock solutions (1.0 × 10^−2^ M) were prepared with doubly distilled water. Only freshly prepared solutions were used.

Silica xerogels doped with iron(III) were obtained by drying in Ethos microwave equipment (Milestone, Italy). Surface area, porosity BET analysis, and BJH pore distribution analysis were carried out with ASAP 2000 (Micromeritics, Norcross, GA, USA). IR spectra of the xerogels in potassium bromide discs (1 mg of xerogel ground with 50 mg of KBr) were recorded with Frontier FT-IR spectrometer (PerkinElmer, Waltham, MA, USA). Absorbance of solutions (l = 1.0 cm) and xerogels water suspensions (l = 0.1 cm) were measured using Lambda 35 spectrophotometer (PerkinElmer, Waltham, MA, USA) equipped with 50 mm integrating sphere (Labsphere, North Sutton, NH, USA). The pH value was measured using with an HI83303 photometer/pH-meter and HI11310 pH electrode (Hanna Instruments, Woonsocket, RI, USA).

### 4.2. Synthesis of Silica Xerogels Doped with Iron(III)

Silica xerogels were obtained using an earlier-developed procedure [22,26,28]: 20.0 mL of 0.05 mol·L^−1^ hydrochloric acid in 50% ethanol solution was added to 10.0 mL of tetraethyl orthosilicate while stirring. To obtain xerogels doped with iron(III), ferric chloride was added to the sol mixture in order to get the final concentration of 1.0 × 10^−3^–5.0 × 10^−2^ M. The wet gels were dried at 800 W microwave irradiation for 10 min in order to evaporate water and to get dry xerogels. The materials were submitted to the microwave irradiation for several short time intervals (30–60 s). The materials were let to cool down for 2–3 min between these drying intervals, so the temperature of the materials did not exceed 80 °C during the drying process.

Obtained xerogel powders were ground and sorted into fractions with different particle sizes using a set of sieves. The fraction of 0.10–0.16 mm sized xerogel particles was used. After this, fractioning xerogels were washed 3 times with 100.0 mL of doubly distilled water and then dried again at 800 W microwave irradiation. Iron(III) content in the washing waters was determined using test-kits (MedEcoTest, Moscow, Russia) and was used to calculate the amount of iron(III) retained in the xerogel.

### 4.3. General Procedure for the SHA–Silica Xerogel Interaction Study

0.10 g of silica xerogel was added to 5.0 mL of solution, containing 3.0 × 10^−3^ M of SHA at different pH and the obtained mixture was shaken (5–60 min). In order to measure the xerogel absorbance, the wet xerogel particles were transferred from the bottom of the vial to the 0.1 cm glass cuvette filled with distilled water. Then, the xerogel absorbance at 450 nm was recorded. The optimal conditions were chosen in order to reach the maximal absorbance.

SHA residual concentration in solution after the interaction with the xerogel was determined. The dependence of iron(III) complexation rate in the xerogel on the SHA residual concentration in solution was used for the determination of the complex stoichiometry and the equilibrium constant.

Calibration curve was constructed using the absorbance of the xerogel after interaction with the SHA standard solutions in the 1.0 × 10^−6^ M–1.0 × 10^−2^ M range. The least squares method was used to obtain the calibration curve. The limit of detection (LOD) was calculated as 3·standard deviation of the blank (n = 3) divided by the slope value. The limit of quantitation was calculated as 3·LOD.

### 4.4. Sample Preparation and Solid Phase Spectrophotometric Determination Procedure

Synthetic urine was prepared as in [29] and consisted of calcium chloride (0.65 g/L), magnesium chloride (0.65 g/L), sodium chloride (4.6 g/L), sodium sulfate (2.3 g/L), trisodium citrate (0.65 g/L), sodium oxalate (0.02 g/L), monopotassium phosphate (2.8 g/L), potassium chloride (1.6 g/L), ammonium chloride (1.0 g/L), urea (25.0 g/L), creatinine (1.1 g/L), and 2% (wt/vol) glucose.

Human urine sample was collected from a healthy volunteer. The research was carried out according to the World Medical Association Declaration of Helsinki, and informed consent was obtained from the subject. Urine sample was diluted with distilled water (1:1 vol).

0.10 g of silica xerogel was added to 5.0 mL of sample solution (pH 3.3) and was shaken for 60 min. The xerogel absorbance was measured at 450 nm. The concentration of SHA in the sample was calculated using the calibration curve.

## Figures and Tables

**Table 1:** Comparison of textural characteristics of silica xerogels doped with iron(III).

	Iron(III) Concentration in Sol, M	Iron(III) Content in Xerogel, % wt	BET Surface Area, m^2^/g	Micropore Area, m^2^/g	Total Pore Volume, cm^3^/g	Micropore Volume, cm^3^/g	Average Pore Diameter, Å
SiFe1	1.0 × 10^−3^ M	0.04	696	50	0.92	0.01	53
SiFe10	1.0 × 10^−2^ M	0.33	551	454	0.24	0.20	18
SiFe50	5.0 × 10^−2^ M	1.74	529	433	0.23	0.19	17

**Table 2:** Analytical characteristics of salicylhydroxamic acid determination.

Xerogel	Time of Contact, Min	Limit of Detection, M (*n* = 3)	Analytical Range, M	Slope, M^−1^
SiFe1	20	1.9 × 10^−4^	5.8 × 10^−4^–3.0 × 10^−3^	92
	60	8.6 × 10^−5^	2.6 × 10^−4^–1.5 × 10^−3^	151
SiFe10	60	9.1 × 10^−6^	2.7 × 10^−5^–1.5 × 10^−3^	1437

**Table 3:** Determination of salicylhydroxamic acid in spiked urine samples using SiFe10 xerogel (*n* = 3, *p* = 0.95).

Sample	Added, M	Found, M	Relative Standard Deviation, %	Recovery, %
Synthetic urine	5.0 × 10^−5^ M	(6.0 ± 0.7) × 10^−5^ M	6.9	120.5
	5.0 × 10^−4^ M	(5.0 ± 0.4) × 10^−4^ M	4.2	99.7
Human urine	5.0 × 10^−5^ M	(5.4 ± 0.8) × 10^−5^ M	9.0	108.6
	5.0 × 10^−4^ M	(4.9 ± 0.4) × 10^−4^ M	5.0	97.4

**Table 4:** Comparison of hydroxamic acids (HA) determination procedures.

Analyte	Sample	Method	Range, mg/L	Limit of Detection, mg/L	Reference
Suberoylanalide hydroxamic acid (Vorinostat)	Plasma	LC-MS	-	0.125 μg/L	[7]
	Pharmaceuticals	Amperometric	1–14 mg/L	0.4	[11]
Deferoxamine	Pharmaceuticals	HPLC Iron(II) complex	10–96 mg/L	6.0	[9]
	Plasma	HPLC	6–56 mg/L	-	[5]
	Urine	HPLC	1–90 mg/L	0.02	[6]
Salicylhydroxamic acid	Pharmaceuticals	Potentiometric, urease inhibition	0.5–7 mg/L	0.1	[10]
	Pharmaceuticals	Spectrophotometric, UV	0.1–50 mg/L	0.03	[12]
	Urine	HPLC	5–95 mg/L	2	[8]
	Pharmaceuticals	Atomic absorption spectrometry	3–31 mg/L	-	[13]
		Spectrophotometry (Cu(II) complex)	1–18 mg/L		
	Urine	Spectrophotometry (Fe(III) complex)	4.2–230 mg/L	1.4	Present work

HPLC—high performance liquid chromatography, LC-MS—liquid chromatography-mass spectrometry.

## Data Availability

Not applicable.

## Supplementary Materials

The following are available online at https://www.mdpi.com/article/10.3390/gels7030143/s1, Table S1: The calculation of the equilibrium constant, Table S2: The interference of albumin, ascorbic acid, and salicylate on the determination of 1.0 × 10^−4^ M SHA.
