# Supplementary material for: Silica Xerogel Doped with Iron(III) as Sensor Material for Salicylhydroxamic Acid Determination in Urine
Source: Gels. 2021 Sep 16;7(3):143. doi: 10.3390/gels7030143 (PMC8482224; doi:10.3390/gels7030143)
Supplement: Supplementary file 1 [file gels-07-00143-s001.zip › gels-1353049-supplementary.pdf]

gels

Table S1. The calculation of the equilibrium constant.

Initial SHA concentration, M	Residual SHA concentration, M ($[H_2L]$)	lg $[H_2L]$	Xerogel absorbance (A_i)	lg ($A_i/(A_{ex}-A_i)$)
$7.5\cdot10^{-5}$	$6.2\cdot10^{-5}$	−4.21	0.0179	−1.64
$1.5\cdot10^{-4}$	$1.3\cdot10^{-4}$	−3.88	0.0377	−1.31
$2.6\cdot10^{-4}$	$2.2\cdot10^{-4}$	−3.65	0.0593	−1.10
$7.5\cdot10^{-4}$	$6.3\cdot10^{-4}$	−3.20	0.1344	−0.70
$1.5\cdot10^{-3}$	$1.3\cdot10^{-3}$	−2.90	0.2320	−0.39
$2.3\cdot10^{-3}$	$1.9\cdot10^{-3}$	−2.73	0.3096	−0.20
$3.0\cdot10^{-3}$	$2.5\cdot10^{-3}$	−2.60	0.3729	−0.06
$5.0\cdot10^{-3}$	$4.1\cdot10^{-3}$	−2.38	0.5075	0.23
$1.0\cdot10^{-2}$	$8.7\cdot10^{-3}$	−2.06	0.6026	0.47
$1.5\cdot10^{-2}$	$1.4\cdot10^{-2}$	−1.85	0.7182	0.92
$2.0\cdot10^{-2}$	$1.9\cdot10^{-2}$	−1.72	0.8051 (A_{ex})	-

SHA—Salicylhydroxamic acid.

Table S2. The interference of albumin, ascorbic acid, and salicylate on the determination of $1.0\cdot10^{-4}$ M SHA.

Interfering substance concentration		SHA found $\cdot10^4$, M	Error, %
Albumin	100 mg/L	1.01	0.6
	500 mg/L	0.92	−7.7
	700 mg/L	0.94	−5.6
Ascorbic acid	$1.0\cdot10^{-4}$ M	1.05	4.6
	$5.0\cdot10^{-4}$ M	1.08	7.5
	$1.0\cdot10^{-3}$ M	0.64	−35.8
Salicylate	$5.0\cdot10^{-4}$ M	1.06	5.7
	$1.0\cdot10^{-3}$ M	1.09	9.2
	$5.0\cdot10^{-3}$ M	1.10	10.3

SHA—Salicylhydroxamic acid.
